# Comparison between 200 mg QD and 100 mg BID oral celecoxib in the treatment of knee or hip osteoarthritis

**DOI:** 10.1038/srep10593

**Published:** 2015-05-27

**Authors:** Chao Zeng, Jie Wei, Hui Li, Tuo Yang, Shu-guang Gao, Yu-sheng Li, Yi-lin Xiong, Wen-feng Xiao, Wei Luo, Tu-bao Yang, Guang-hua Lei

**Affiliations:** 1 Department of Orthopaedics, Xiangya Hospital, Central South University, Changsha, Hunan Province, China, 410008; 2 Department of Epidemiology and Health Statistics, School of Public Health, Central South University, Changsha, Hunan Province, China, 410008

## Abstract

This network meta-analysis aimed to investigate the effectiveness and safety of 100 mg BID and 200 mg QD oral celecoxib in the treatment of OA of the knee or hip. PubMed, Embase and Cochrane Library were searched through from inception to August 2014. Bayesian network meta-analysis was used to combine direct and indirect evidences on treatment effectiveness and safety. A total of 24 RCTs covering 11696 patients were included. For the comparison in between the two dosage regimens, 100 mg BID oral celecoxib exhibited a greater probability to be the preferred one either in terms of pain intensity or function at the last follow-up time point. For total gastrointestinal (GI) adverse effects (AEs), both of the two dosage regimens demonstrated a higher incidence compared to the placebo group. Further analyses of GI AEs revealed that only 200 mg QD was associated with a significantly higher risk of abdominal pain when compared with placebo. Furthermore, 100 mg BID showed a significantly lower incidence of skin AEs when compared with 200 mg QD and placebo. Maybe 100 mg BID should be considered as the preferred dosage regimen in the treatment of knee or hip OA.

Osteoarthritis (OA) is the arthritis from with the highest prevalence in the world, affecting about 40% of the total population aged over 70^1^. In America, at least 20 million people suffered from OA, and this figure is expected to double over the next 20 years^2^. Recently, two evidence-based guidelines were developed by Osteoarthritis Research Society International (OARSI) and American Academy of Orthopaedic Surgeons (AAOS), in which some commonly used pharmacologic treatments, such as chondroitin, glucosamine and intra-articular hyaluronic acid, were regarded as “uncertain appropriateness” or “not recommended”^3^,^4^. Fortunately, non-steroidal anti-inflammatory drugs (NSAIDs) were approved by both of them for the management of knee OA. However, out of the reason of inhibiting both cyclo-oxygenase-1 (COX-1) and COX-2 to varying degrees, traditional NSAIDs were criticized for their side-effects, such as gastrointestinal (GI) reactions. Approval by American Food and Drug Administration (FDA) in December 1998, celecoxib was the first and quickly became the most frequently prescribed specific inhibitor of COX-2. Due to an increased risk of cardiovascular (CV) side effects, rofecoxib and valdecoxib have been withdrawn from the market^5^,^6^. Lumiracoxib, etoricoxib and etodolac have not even been approved by FDA yet because of concerns on adverse effects (AEs). Celecoxib, thus turned into the only selective NSAID currently available in America^7^.

According to the official instructions of celecoxib, 200 mg QD and 100 mg BID are the two recommended dosage regimens for the treatment of OA. A few years ago, Williams *et al.* conducted two randomized controlled trials (RCTs) and demonstrated that celecoxib 200 mg daily and 100 mg twice a day were equally effective and well tolerated in patients with knee OA^8^,^9^. This finding was subsequently confirmed by Stengaard-Pedersen *et al.*^10^ Out of the concern of convenience, especially for old patients, doctors have been generally in favor of 200 mg QD in all these years. However, peak plasma of celecoxib concentration occurs after 2 to 4 hours and its half-life occurs about 11 hours^11^, which means that 100 mg BID could possibility lead to a more steady plasma drug concentration and a lower peak plasma concentration. It remains interesting and meaningful to figure out whether 100 mg BID could result in better efficacy and lower incidence of side effects in clinical practice. If this speculation can be confirmed, the conventional view and practice will be reversed.

Classical meta-analysis was limited due to the lack of multiple comparisons. Bayesian network meta-analysis is a method combining all available direct (studies compared 200 mg QD with 100 mg BID directly) and indirect evidences (studies compared 200 mg QD with 100 mg BID via the placebo group) on the relative treatment effects, enabling a unified; and coherent analysis of all RCTs^12^,^13^,^14^,^15^. Network meta-analysis not only can increase the statistical power by combining evidence from both direct and indirect comparisons, but also can examine the relative effects of different interventions that have few comparisons or never have been compared directly^16^. In our case, the use of both direct and indirect evidence can improve the estimate accuracy by shortening the width of the confidence intervals in contrast to the use of direct estimate alone^17^. With the accumulation of recent evidences, this study performed a network meta-analysis on RCTs which investigated the efficacy and safety of 200 mg QD and 100 mg BID oral celecoxib in the management of knee or hip OA.

## Methods

### Literature search

Searches of electronic databases of PubMed, Embase and Cochrane Library were done using a series of logic combination of keywords and text words related to OA to identify interested interventions and RCTs (see web extra appendix 1) dated up to August 2014. The database search was then supplemented by subsequent periodic scrutiny of unpublished and ongoing studies from the following websites: Current Controlled Trials (http://www.controlledtrials.com/), ClinicalTrials.gov (http://www.clinicaltrials.gov/) and the World Health Organization International Clinical Trials Registry (http://apps.who.int/trialsearch/Default.aspx). In addition, references of the retrieved papers and reviews were also manually identified.

### Study selection

Papers meeting the following criteria were included in this meta-analysis: (1) large RCTs (at least 100 patients per arm)^18^; (2) studies on patients with knee or hip OA (3) studies containing at least two of the following eligible treatments: 200 mg QD oral celecoxib, 100 mg BID oral celecoxib and the placebo group; (4) studies reporting the pain, function or side effects outcomes of patients. Secondary studies, including some combined data analysis of RCTs, were excluded.

### Quality assessment

The modified oxford score^19^,^20^, a scale ranged from 0 to 7 according to the descriptions of randomization, the concealment allocation, the blinding method and the reporting of participant withdrawals, were used to measure the methodological quality of included studies.

### Outcome measure

The primary outcome of this study was the effectiveness of pain relief and function improvement from the baseline to the end of the treatment by applying 200 mg QD or 100 mg BID oral celecoxib therapy for knee or hip OA. If a study reported multiple pain scales, the highest one on the hierarchy of pain scale related to outcomes was adopted, as described by Jüni and colleagues^21^. The function subscale of Western Ontario and McMaster Universities Arthritis Index (WOMAC) was referenced to assess the function improvement. If a study did not measure or report the WOMAC function, WOMAC total, Lequesne Index or other functional measurement scales were used for analysis instead. Standard mean differences (SMD) were used to identify the difference between different treatment arms for quantitative data. A negative value of SMD indicates a better effect in pain relief and function improvement after treatment.

Safety and tolerability were also examined by comparing the number of patients who withdrew due to AEs and who suffered from serious AEs (SAEs) and the incidence of common reported AEs. No standard was set in this study for identifying SAEs; they were defined by their original research. AEs were classified into eight kinds of events in order to summarize their variety in all included studies: GI AEs, CV AEs, central nervous system (CNS) AEs, musculoskeletal AEs, infections, skin AEs and peripheral edema. Below is the detailed description of the AEs classification:

- GI AEs: dyspepsia, diarrhea, abdominal pain, nausea, constipation, flatulence, vomit.
- CV AEs: chest pain, hypertension, thrombotic CV AE, stoke, myocardial infarction, congestive heart failure, CV events excluding chest pain.
- CNS AEs: headache, dizziness, insomnia, depression, nervousness, anorexia.
- Musculoskeletal AEs: arthralgia, back pain, myalgia, sciatica.
- Infections: upper respiratory infection, nasopharyngitis, influenza, urinary tract infection, sinusitis, bronchitis, rhinitis, pharyngitis.
- Skin AEs: rash, pruritus, exanthema, erythema, itching, dry skin, skin irritation, urticarial, allergic dermatitis.
- Peripheral edema.

Meanwhile, six most common reported GI AEs (abdominal pain, dyspepsia, diarrhea, nausea, constipation, flatulence) were analyzed separately due to their high incidence during the treatment. Odds ratios (OR) were calculated to determine the difference between compared groups.

### Statistical analysis

A Bayesian random effect model of network meta-analysis was used to compare the overall effect size among different celecoxib dosage regimens and placebo for knee and hip OA. The advantage of network meta-analysis is that it combines the evidences of both direct and indirect comparisons in all primary trials^16^. The statistical method used in the present study was described in our previous researches^22^,^23^. Bayesian method let the prior probability distribution taking into account the prior information. We used vague prior (mean 0, variance 10000) distributions throughout, allowing the data to drive inferences. Markov Chain Monte Carlo methods was used through WinBUGS software (version 1.4.3, MRC Biostatistics Unit, Cambridge, UK) to estimate posterior densities for unknown variables according to some high-quality studies^15^,^24^,^25^. The random effects model was adopted rather than the fixed effects models as the most appropriate and conservative analysis to account for differences among trials. For random effects, we made the assumption of homogeneous variance. Three Markov chains ran simultaneously with different initial values which were chosen arbitrarily for convergence. A total of 50,000 simulations were generated for each of the three sets of initial values, and the first 10,000 simulations were discarded due to the burn-in period. The WinBUGS codes of random effect models for multi-arm trials are available at http://www.mtm.uoi.gr/ and http://www.bristol.ac.uk/social-community-medicine/projects/mpes/ (WinBUGS codes for network meta-analysis see Appendix 2). The overall effect sizes (ORs or SMDs) were generated from the median of the posterior distribution. The 2.5th and 97.5th percentiles of the posterior distribution were considered as the lower and upper limit of the traditional corresponding 95% credible interval (95%CI) respectively. A significant difference could be identified by 95%CI which did not include 1 for OR, or 0 for SMD. Inconsistency is defined by the differences between direct and indirect effect estimates for the same comparison, and was evaluated in this study by using the ratio of two odds rations (*RoR*) from direct and indirect evidences in one loop. A *RoR* value closer to 1 suggests that the two estimations are consistent to each other. Loops with the lower 95%CI limit of *RoR* value do not reach the 1 line represent statistically significant inconsistency^26^. The fit of the model to data can be measured by calculating the posterior mean residual deviance. If the mean of the residual deviance is close to the number of data points of the model, it indicates that this model fits the data adequately^27^. Network meta-analysis can also generate rankings for all evaluated treatments based on the level of effectiveness according to their posterior probabilities (first best, second best, third best, etc.). The probability values were summarized and reported as the surface under the cumulative ranking (SUCRA). SUCRA is equal to 100% for the best treatment, and 0% for the worst treatment^26^,^28^. The classic pairwise meta-analysis was also conducted. Heterogeneity was tested by Q statistics (P ≤ 0.05 was considered heterogeneous) and I^2^ statistics, which measures the percentage of the total variation across various studies (I^2^ ≥ 50% was considered heterogeneous). Publication bias was evaluated by Begg’s test, and a P value equal to or less than 0.05 represents the existence of publication bias^29^.

All statistical analyses were implemented by using WinBUGS software (version 1.4.3, MRC Biostatistics Unit, Cambridge, UK), R version 3.0.2 (The R Foundation for Statistical Computing) and STATA software (version 11.0, StataCorp, College Station, TX).

## Results

### Study selection and characteristics

**Figure 1:**
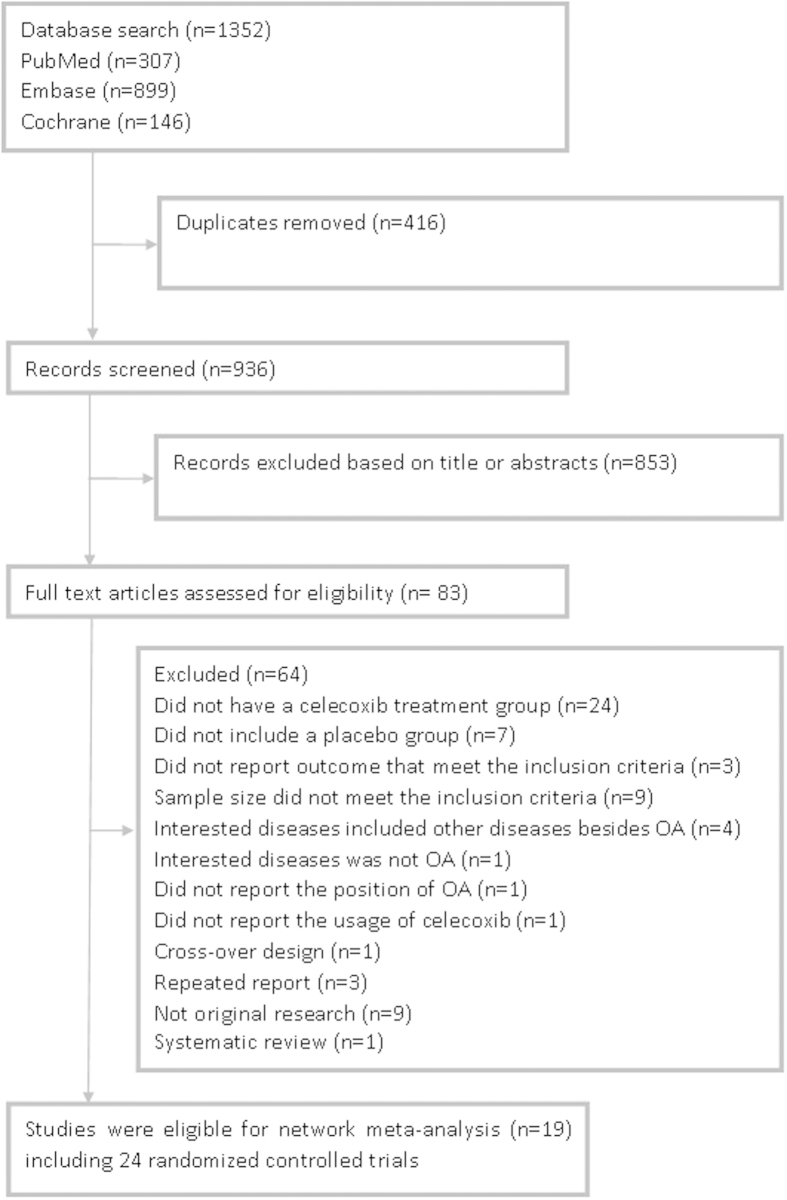
Summary of studies identification and selection.

**Figure 2:**
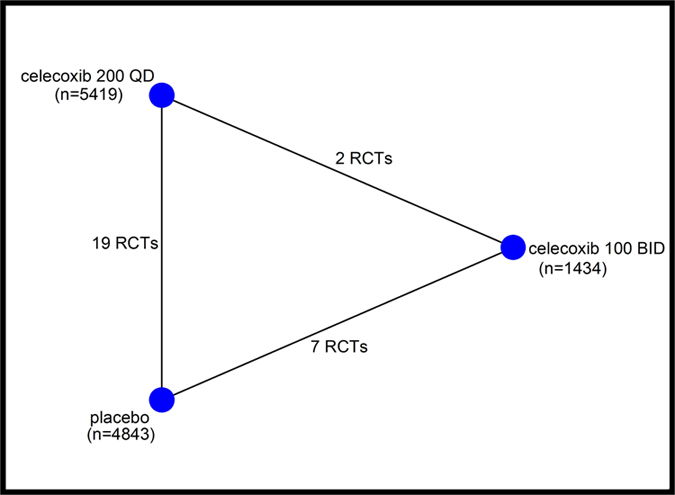
Structure of network formed by interventions and their direct comparisons. The lines between treatment nodes indicate the direct comparisons made within randomized trials.

Figure 1 summarizes the results of evidence search and selection. Finally, 19 studies (24 RCTs)^8^,^9^,^30^,^31^,^32^,^33^,^34^,^35^,^36^,^37^,^38^,^39^,^40^,^41^,^42^,^43^,^44^,^45^,^46^ were included in this meta-analysis. All 24 RCTs were multicenter, randomized, double-blind, placebo-controlled trials assessing the efficacy and safety of oral celecoxib (100 mg BID or 200 mg QD) for the patients with knee or hip OA. The characteristics and the results of methodological quality assessment of the included studies were presented in Table 1. Fourteen trials included patients with OA of the knee only, eight trials included patients with OA of either the hip or knee, and two trials included patients with OA of the hip only. In summary, the total data available for network meta-analysis involved 11696 patients (1434 participants in 100 mg BID group, 5419 in 200 mg QD and 4843 in placebo) with either the knee or hip OA. Figure 2 showed the network structure of the comparisons in this study.

### Effects of joint pain

**Figure 3:**
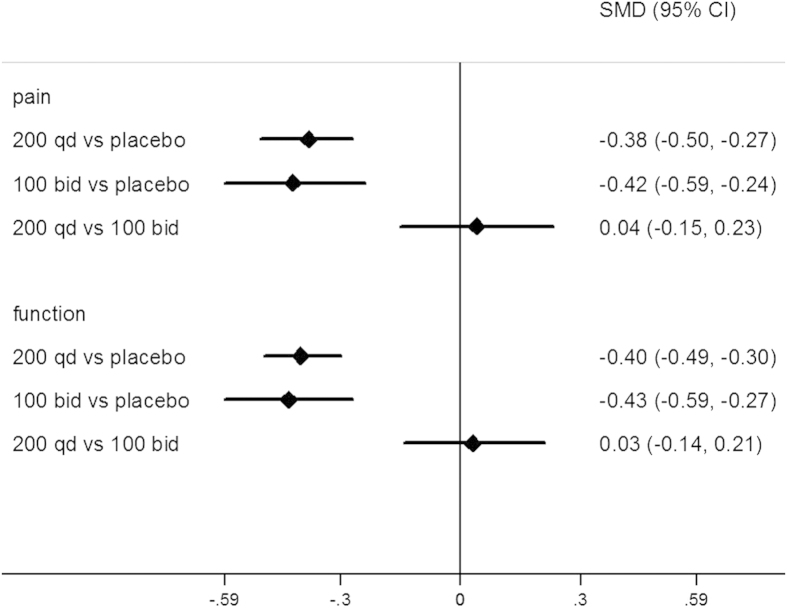
Network meta-analysis estimates-standard mean difference (SMD) of pain relief and function improvement for three compared groups.

**Figure 4:**
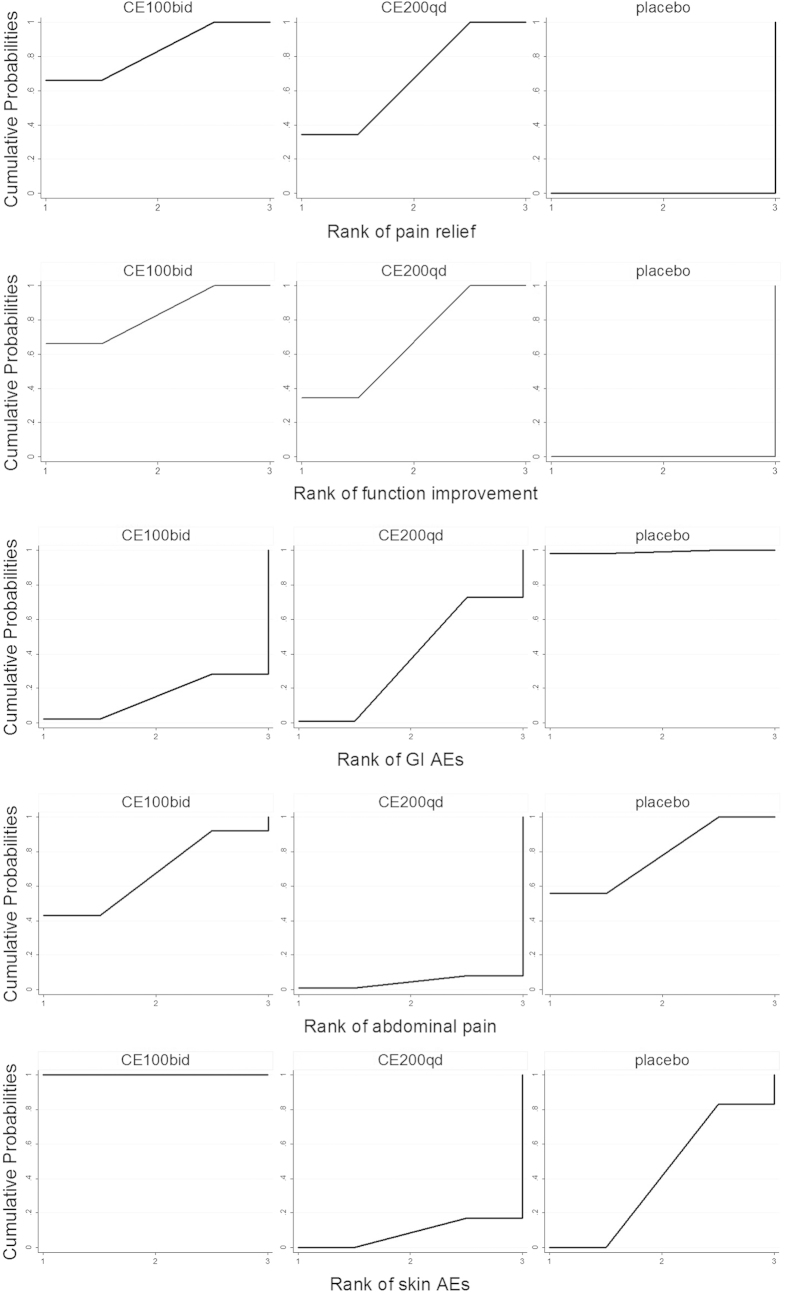
Rankings for three treatments. Graph displays distribution of probabilities for each treatment. X-axis represents the possible rank of each treatment (from the best rank to worse according to the outcomes), Y-axis represents the cumulative probability for each treatment to be the best option, among the best two options, among the best three options, and so on.

The results of network meta-analysis and pairwise meta-analysis were reported in Table 2. 200 mg QD oral celecoxib achieved a significantly lower pain intensity compared to the placebo group (SMD: −0.38, 95%CI: −0.50 to −0.27), and 100 mg BID oral celecoxib achieved a significantly better effect of pain management compared to the placebo group (SMD: −0.42, 95%CI: −0.59 to −0.24). However, there was no significant difference between the 200 mg QD and 100 mg BID group in terms of pain intensity (SMD: 0.04, 95%CI: −0.15 to 0.23) (Fig. 3). No evidence of inconsistency between direct and indirect estimates was found in this network meta-analysis. Evaluation of the goodness of fit indicated an adequate fit with a posterior mean residual deviance of 41.2 (40 data points). The probability distribution of each treatment for this outcome was shown in Fig. 4. 100 mg BID exhibited the largest probability to be the best treatment (83%) compared to 200 mg QD (67%) and the placebo group (0%). Such finding was further supported by the results of pairwise meta-analysis (Table 2). Significant evidence of heterogeneity was only observed in the comparison between 200 mg QD and the placebo group (*p* = 0.00, I^2^ = 80%). There was no publication bias among various studies.

### Effects of joint function

Table 2 also showed the outcomes of network meta-analysis in terms of function improvement. 200 mg QD oral celecoxib was significantly more effective compared to the placebo group (SMD: −0.40, 95%CI: −0.49 to −0.30), and so was 100 mg BID oral celecoxib (SMD: −0.43, 95%CI: −0.59 to −0.27). However, there was no significant difference between the 200 mg QD and the 100 mg BID group (SMD: 0.03, 95%CI: −0.14 to 0.21) (Fig. 3). No evidence of inconsistency between direct and indirect estimates was found in this network meta-analysis. Evaluation of the goodness of fit indicated an adequate fit with a posterior mean residual deviance of 39.77 (38 data points). The probability distribution of each treatment for this outcome was shown in Fig. 4. 100 mg BID exhibited the largest probability to be the best treatment (83%) compared to 200 mg QD (67%) and the placebo group (0%). Such finding was further supported by the results of pairwise meta-analysis (Table 2). Significant evidence of heterogeneity was only observed in the comparison between 200 mg QD and the placebo group (*p* = 0.00, I^2^ = 75%). There was no publication bias among various studies.

### Tolerability and adverse effects

**Figure 5:**
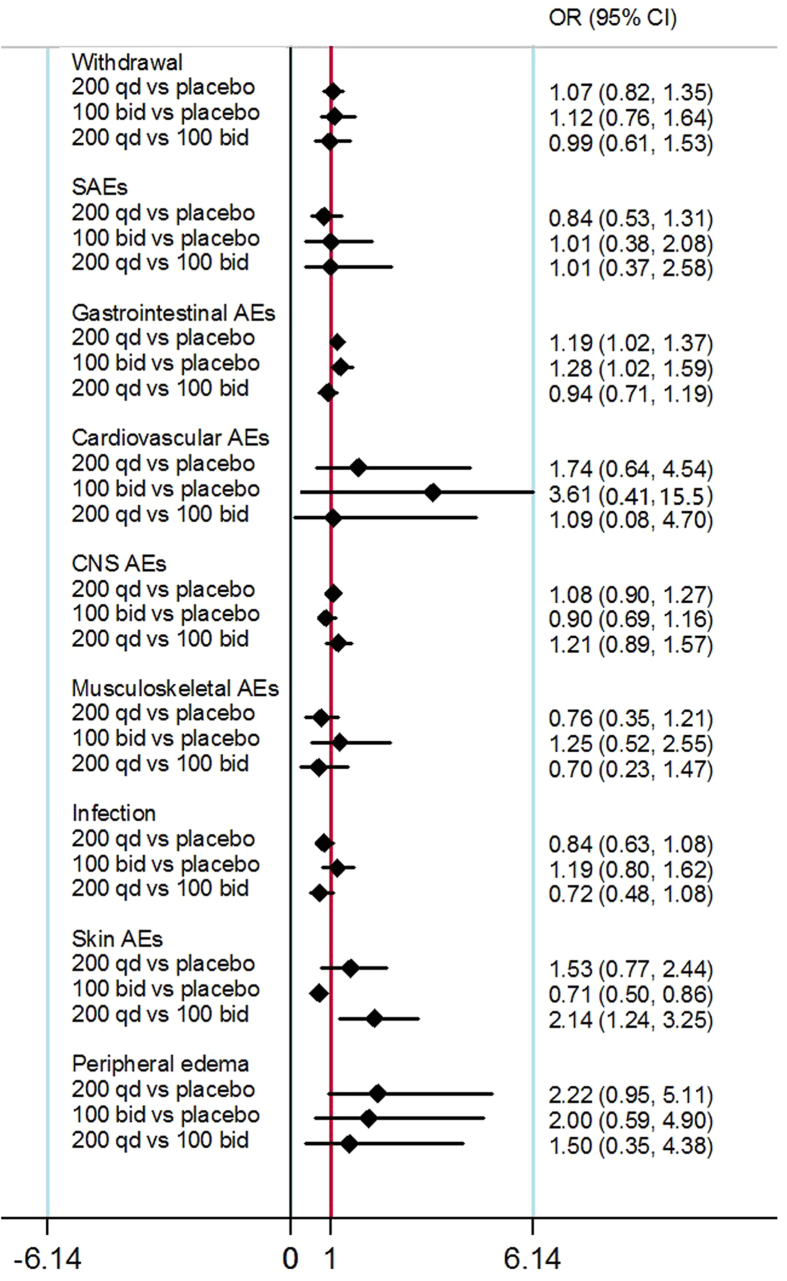
Network meta-analysis estimates-odds ratios (OR) of eight kinds of AEs for three compared groups.

The results of tolerability and AEs (including withdrawal due to AE and other eight kinds of AEs) were reported in Table 2. For GI AEs, both 200 mg QD (OR: 1.19, 95%CI: 1.02 to 1.37) and 100 mg BID (OR: 1.28, 95%CI: 1.02 to 1.59) showed a higher incidence compared to the placebo group, but there was no significant difference between the 200 mg QD and the 100 mg BID group (OR: 0.94, 95%CI: 0.71 to 1.19) (Fig. 5). No evidence of inconsistency between direct and indirect estimates was found in this network meta-analysis. Evaluation of the goodness of fit indicated a moderate fit with a posterior mean residual deviance of 33.97 (38 data points). The probability distribution of each treatment for this outcome was shown in Fig. 3. 200 mg QD (1%) and 100 mg BID (2%) achieved a similar ranking compared to the placebo group (98%). There was no evidence of significant heterogeneity and publication bias in the comparison among 200 mg QD, 100 mg BID and the placebo group for GI AEs.

**Figure 6:**
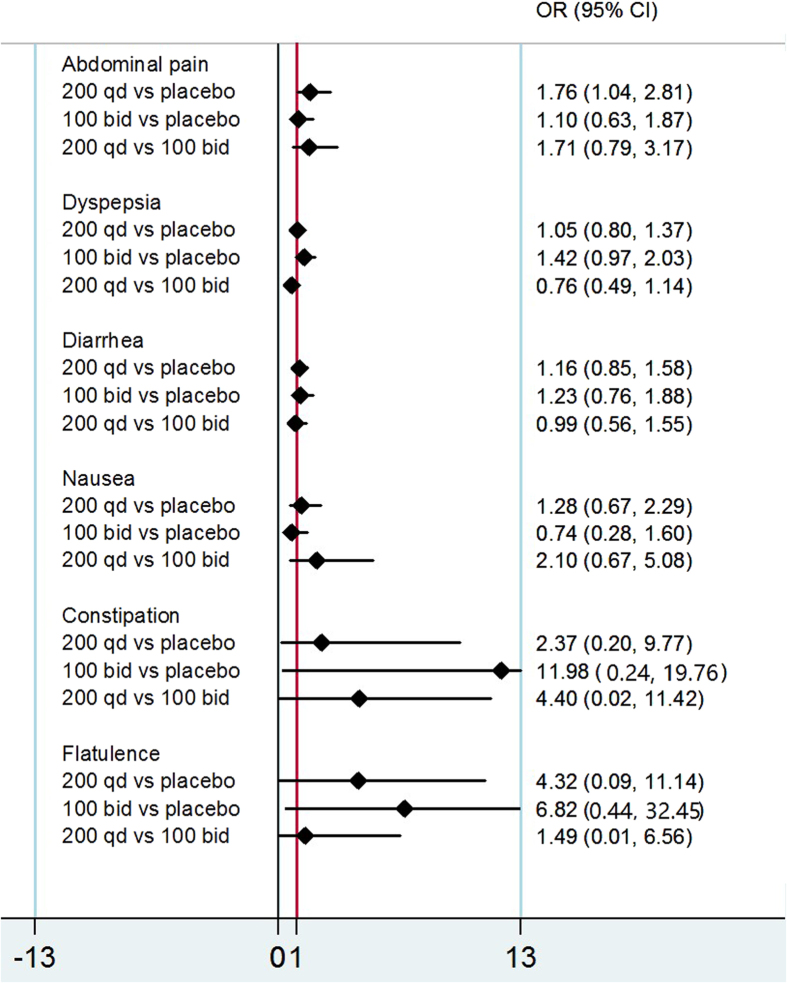
Network meta-analysis estimates-odds ratios (OR) of six kinds of GI AEs for three compared groups.

Six kinds of GI AEs (including abdominal pain, dyspepsia, diarrhea, nausea, constipation and flatulence) were analyzed separately (Fig. 6). It is noteworthy that 200 mg QD oral celecoxib showed a higher incidence of abdominal pain compared to the placebo group (OR: 1.76, 95%CI: 1.04 to 2.81). No evidence of inconsistency between direct and indirect estimates was found in this network meta-analysis. Evaluation of the goodness of fit indicated a moderate fit with a posterior mean residual deviance of 14.76 (21 data points). The probability distribution of each treatment for this outcome was shown in Fig. 3. 100 mg BID is likely to be a better treatment (43%) compared to 200 mg QD (1%). Such finding was further supported by the results of pairwise meta-analysis (Table 2). No evidence of significant heterogeneity and publication bias existed in the comparison among various studies.

For Skin AEs, 100 mg BID showed a significantly lower incidence compared to the placebo group (OR: 0.71, 95%CI: 0.50 to 0.86), while 200 mg QD showed a significantly higher incidence compared to the 100 mg BID group (OR: 2.41, 95%CI: 1.24 to 3.25) (Fig. 5). No evidence of inconsistency between direct and indirect estimates was found in this network meta-analysis. Evaluation of the goodness of fit indicated an adequate fit with a posterior mean residual deviance of 11.27 (10 data points). The probability distribution of each treatment for this outcome was shown in Fig. 4. 100 mg BID is likely to be a better treatment (100%) compared to 200 mg QD (0%) and the placebo group (0%). However, the pairwise meta-analysis did not support any significant difference in terms of skin AEs (Table 2). No evidence of significant heterogeneity and publication bias existed in the comparisons among various studies.

For all the rest kinds of AEs, no evidence of significant difference was observed in this network meta-analysis. The probability distribution of each treatment for non-significant AEs was shown in Appendix 3, and all the forest plots of pairwise meta-analysis were presented in Appendix 4.

## Discussion

Several meta-analyses and systematic reviews on celecoxib have been published^24^,^47^,^48^,^49^,^50^,^51^,^52^,^53^,^54^. In 2002, Deeks and colleagues included 9 trials and examined the efficacy and safety of celecoxib for OA and rheumatoid arthritis (RA). Their findings showed that celecoxib is effective and improved the GI safety and tolerability compared to other NSAIDs^48^. More recently, Essex and colleagues combined 89 RCTs without restricting diseases and provided safety information on the usage of celecoxib^48^. However, a meta-analysis on 31 company clinical trial reports suggested that celecoxib had fewer discontinuations for any cause or for lack of efficacy, fewer SAEs, and less nausea, but more dyspepsia, diarrhoea, odema and GI AEs^49^. 4 meta-analyses or systematic reviews specifically aimed at assessing the CV safety of celecoxib^24^,^50^,^51^,^52^. In 2011, a network meta-analysis conducted by Trelle and colleagues indicated that little evidence existed to support the safety of any investigated drugs, including celecoxib, in CV terms^6^. A previous meta-analysis also showed that selective COX-2 inhibitors were associated with a moderate increase in the risk of vascular events, and meanwhile, significant events with higher daily doses were found for celecoxib^50^. Nevertheless, another two analyses did not demonstrate an increased CV risk with celecoxib relative to placebo, and suggested that the commonly used doses of celecoxib may not increase the risk^51^,^52^. As for GI AEs, the safety of celecoxib was proved by another two studies^53^,^54^. However, none of the previous meta-analyses and systematic reviews specifically aimed to examine the efficacy and safety of celecoxib in the treatment of OA; therefore, the doses were generally ranged from 25 to 800 mg daily, which made it difficult for decision-making in clinical practice. Treatments related to AEs were associated with various dosages of celecoxib^55^.

As described in the introduction of this study, 3 RCTs suggested that there was no difference between celecoxib 200 mg QD and 100 mg BID in efficacy or safety for the management of OA, providing flexibility to patients and physicians in choosing a dosing regimen^8^,^9^,^10^. However, the findings of this network meta-analysis are contrary to their conclusions. This research recommended the use of 100 mg BID oral celecoxib in the management of patients with knee or hip OA, as it is more likely to provide a better effect in pain relief and function improvement. As a speculation about the cause of this finding, 100 mg BID may lead to a more steady plasma drug concentration, although this hypothesis remains highly speculative. This result was more or less in line with another finding, which indicated that the once-daily dosing of celecoxib resulted in a less sustained blood pressure effect than the twice-daily dosing^56^. This network meta-analysis also suggested that 100 mg BID was safer than 200 mg QD in terms of abdominal pain and skin AEs, which probably be explained by the higher peak plasma concentration of 200 mg QD. Peak plasma levels of celecoxib are dose proportional up to 200 mg BID^57^. Considering that the peak plasma concentration occurs after 2 to 4 hours^11^, 200 mg QD shall certainly lead to a higher peak plasma concentration than 100 mg BID, which may be associated with the significant difference in those two AEs.

However, Solomon and colleagues conducted a pooled analysis of 6 RCTs and came to a noteworthy speculation that 400 mg QD celecoxib might be safer than 200 mg BID because the former regimen was associated with a shorter duration of exposure to susceptible atherosclerotic tissue^58^. This speculation was based on the condition of the 1.5 hours of half-life after a single oral celecoxib, as the authors indicated. However, the correct half-life was actually 11.5 hours rather than 1.5 hours, which was clearly presented in the original reference cited by the authors^59^. Anyway, this result is not incompatible with our finding that 100 mg BID is a more preferred dosage regimen than 200 mg QD in the treatment of knee or hip OA.

The findings of this network meta-analysis are extremely important to clinic practice. On one hand, this study provided evidence to support the two evidence-based guidelines (OARSI and AAOS)^3^,^4^ and FDA for the recommended use of celecoxib in the treatment of OA. On the other hand, this study suggested that 100 mg BID oral celecoxib is more likely (higher probability) to be a better option of dosage regimen compared to 200 mg QD in terms of both pain relief and function improvement. What is important is that 100 mg BID also has a better safety than 200 mg QD in terms of abdominal pain and skin AEs. Some may argue, however, that celecoxib is more expensive than traditional NSAIDs, but it cannot be denied that celecoxib is probably as effective as other traditional NSAIDs and significantly improved the GI safety and tolerability^47^. Medical costs actually associated with the treatment of common AEs and disabling conditions, which of course might impose an economic burden to patients and healthcare systems. Therefore, the use of celecoxib may result in a lower medical cost^60^. Above all, even though 200 mg QD was more convenient than 100 mg BID, especially for old people, this study recommended the dosage regimen of 100 mg BID for the treatment of knee or hip OA. The conventional view and clinical practice should be reversed.

A single study is impossible to solve all the problems pertinent to the evaluation of oral celecoxib for knee or hip OA. Considering the small number of direct evidence comparing 200 mg QD with 100 mg BID, further high quality RCTs of direct comparison, especially industry independent trials, are needed. Additional questions to be addressed include the determination of the best treatment duration (shortest in the case of effective). Furthermore, the follow-up time of the subsequent studies also needs to be extended in order to see whether the effects may diminish and whether there were delayed AEs, especially for CV AEs.

As far as we know, this is the first network meta-analysis on two different official dosage regimens of oral celecoxib (200 mg QD and 100 mg BID) for the treatment of knee or hip OA. It combined evidences from both direct and indirect comparisons while fully preserving randomisation for evaluating the relative effectiveness in pain relief, function improvement and safety. In addition, this is also the first meta-analysis that restricted subjects to OA patients for examining the effect and safety of oral celecoxib. Furthermore, a comprehensive literature search was performed in several major databases and sources to cover as many eligible trials as possible, so the chance of missing any relevant trial was fairly low. With the pre-stated inclusion criteria on the sample size threshold, only large-scale RCTs were included in this network meta-analysis, which enhanced the robustness of the results^18^.

Nevertheless, the limitations of this study should not be ignored. Firstly, variations in duration and final follow-up time point might contribute to the evidence of significant heterogeneity, particularly for the possible duration-response patterns which could affect the performance of celecoxib. Fortunately, no obvious evidence of inconsistency was observed in this network meta-analysis. Secondly, all of the included trials measured the pain or function parameters at a time point too shortly after the completion of the treatment courses. It is uncertain whether these effects might diminish over a period of time. Thirdly, none of the included trials was industry independent trial, so there is a possibility that the effect size was overestimated^61^. Fourthly, this study cannot reach a conclusion that is fully applicable to hip OA because of the limited number of included trials with hip OA patients only. Last but not the least, most of the studies only recorded AEs at the end of the treatment (only two studies postponed the measurement to 30 day later^31^,^43^), so it is unclear whether delayed AEs would emerge.

## Conclusion

This network meta-analysis indicated that 200 mg QD and 100 mg BID oral celecoxib are both effective in pain relief and function improvement for the management of knee or hip OA. Overall, 100 mg BID exhibited a greater probability of being the preferred regimen in pain relief and function improvement, and it also showed a significantly lower risk of abdominal pain and skin AEs when compared with 200 mg QD, so maybe 100 mg BID oral celecoxib should be considered as the recommend dosage regimen in the treatment of knee or hip OA. Further RCTs are needed to confirm this result.

## Additional Information

**How to cite this article**: Zeng, C. *et al.* Comparison between 200 mg QD and 100 mg BID oral celecoxib in the treatment of knee or hip osteoarthritis. *Sci. Rep.* **5**, 10593; doi: 10.1038/srep10593 (2015).

## Figures and Tables

**Table 1:** Characteristics and methodological assessment of the included 24 RCTs.

**Study**	**Location of OA**	**Sample size (female %)**∮	**Mean age (years)**	**Duration (weeks)**	**Follow-up time point (weeks)**	**Randomised method**	**Allocating concealment**	**Blinding method**	**Withdrawal**	**Total score**
Celecoxib 100 mg BID vs. 200 mg QD vs. placebo										
Williams 2000	Knee	231(67)/223(66)/232(67)	63/63/63	6	2, 6	1	2	2	1	6
Williams 2001	Knee	243(69)/231(69)/244(73)	62/61/61	6	2, 6	1	2	2	1	6
Celecoxib 100 mg BID vs. placebo										
Bensen 1999	Knee	197(73)/203(75)	62/62	12	2, 6, 12	2	2	2	1	7
Conaghan 2013	Knee	233(67)/227(66)	61/62	12	2, 6, 9, 12	2	2	2	1	7
McKenna 2001	Knee	201(68)/200(66)	62/60	6	2, 6	1	0	1	1	3
Rother 2007	Knee	132(62)/127(63)	62/63	6	2, 4, 6	2	0	1	1	4
Zhao 1999	Knee	197(73)/204(75)	62/62	12	2, 6, 12	1	0	1	1	3
Celecoxib 200 mg QD vs. placebo										
Bingham 2007 (1)	Knee or hip	241(70)/127(65)	63/63	12	2, 4, 8, 12	1	0	2	1	4
Bingham 2007 (2)	Knee or hip	247(62)/117(65)	62/61	12	2, 4, 8, 12	1	0	2	1	4
Clegg 2006	Knee	318(67)/313(64)	59/58	24	4, 8, 16, 24	2	0	2	1	5
DeLemos 2011	Knee or hip	202(65)/200(69)	60/59	12	1, 2, 3, 6, 9, 12	1	2	2	1	6
Fleischmann 2006	Knee	444(67)/231(66)	61/62	13	2, 4, 8, 13	1	0	2	1	4
Hochberg 2011 (1)	Knee	242(61)/124(66)	62/62	12	6, 12	2	0	2	1	5
Hochberg 2011 (2)	Knee	244(63)/122(63)	62/62	12	6, 12	2	0	2	1	5
Kivitz 2001	Hip	207(65)/218(67)	62/64	12	2, 6, 12	2	0	2	1	5
Lehmann 2005	Knee	420(68)/424(72)	63/62	13	2, 4, 13	2	0	2	1	5
Lisse 2001*	Knee or hip	191(68)/188(66)	75/74	12	2, 12	1	0	1	1	3
Schnitzer 2011	Hip	419(61)/416(61)	62/61	13	4, 8, 13	2	2	2	1	7
Sheldon 2005	Knee	393(63)/382(61)	60/61	13	2, 4, 8, 13	1	0	2	1	4
Smugar 2006 (1)	Knee or hip	456(68)/150(69)	62/62	6	2, 4, 6	1	0	1	1	3
Smugar 2006 (2)	Knee or hip	460(66)/151(68)	62/63	6	2, 4, 6	1	0	1	1	3
Tannenbaum 2004	Knee	481(69)/243(67)	64/65	13	2, 4, 8, 13	1	0	2	1	4

^∮^data was extracted from the baseline;

^*^data was combined from three RCTs.

**Table 2:** Results of network meta-analysis and pairwise meta-analysis.

Ooutcomes	Celecoxib 200 mg QD vs. placebo				Celecoxib 100 mg BID vs. placebo				Celecoxib 200 mg QD vs. 100 mg BID			
	NM,SMD/OR (95%CI)	PM,SMD/OR (95%CI)	I^2^	PB	NM,SMD/OR (95%CI)	PM,SMD/OR (95%CI)	I^2^	PB	NM,SMD/OR (95%CI)	PM,SMD/OR (95%CI)	I^2^	PB
Pain	−0.38(−0.50,−0.27)	−0.39(−0.49,−0.29)	80%	0.24	−0.42(−0.59,−0.24)	−0.43(−0.51,−0.35)	32%	0.71	0.04(−0.15,0.23)	0.04(−0.08,0.17)	15%	—
Function	−0.40(−0.49,−0.30)	−0.40(−0.49,−0.31)	75%	0.28	−0.43(−0.59,−0.27)	−0.45(−0.54,−0.36)	40%	0.81	0.03(−0.14,0.21)	0.04(−0.08,0.17)	15%	—
Tolerability and AEs												
Withdrawal due to AEs	1.07(0.82,1.35)	1.06(0.88,1.26)	36%	0.65	1.12(0.76,1.64)	1.05(0.70,1.56)	48%	1.00	0.99(0.61,1.53)	0.99(0.53,1.86)	0%	
Serious AEs	0.84(0.53,1.31)	0.78(0.54,1.12)	0%	0.24	1.01(0.38,2.08)	1.03(0.56,1.90)	0%	0.03	1.01(0.37,2.58)	5.26(0.25,110.18)	—	—
Gastrointestinal AEs	1.19(1.02,1.37)	1.19(1.04,1.37)	0%	0.58	1.28(1.02,1.59)	1.25(1.01,1.54)	0%	1.00	0.94(0.71,1.19)	0.82(0.59,1.16)	26%	—
Abdominal pain	1.76(1.04,2.81)	1.78(1.12,2.83)	0%	0.46	1.1(0.63,1.87)	1.04(0.61,1.76)	0%	1.00	1.71(0.79,3.17)	1.57(0.26,9.48)	—	—
Dyspepsia	1.05(0.8,1.37)	1.06(0.82,1.38)	0%	0.92	1.42(0.97,2.03)	1.29(0.90,1.84)	0%	0.23	0.76(0.49,1.14)	0.62(0.32,1.23)		—
Diarrhea	1.16(0.85,1.58)	0.98(0.73,1.30)	0%	0.21	1.23(0.76,1.88)	1.31(0.83,2.06)	0%	0.76	0.99(0.56,1.55)	0.83(0.38,1.79)	33%	—
Nausea	1.28(0.67,2.29)	1.32(0.85,2.05)	39%	0.81	0.74(0.28,1.6)	0.62(0.33,1.15)	0%	0.73	2.1(0.67,5.08)	1.04(0.15,7.45)	—	—
Constipation	2.37(0.2,9.77)	1.31(0.57,3.01)	0%	0.30	11.98(0.24,19.76)	1.31(0.47,3.67)	5%	1.00	4.4(0.02,11.42)	—	—	—
Flatulence	4.32(0.09,11.14)	1.20(0.40,3.59)	0%	1.00	6.82(0.44,32.45)	1.93(0.79,4.68)	0%	0.73	1.49(0.01,6.56)	—	—	—
Cardiovascular AEs	1.74(0.64,4.54)	1.13(0.68,1.86)	0%	0.25	3.61(0.41,15.55)	1.88(0.51,6.90)	0%	1.00	1.09(0.08,4.7)	—	—	—
CNS AEs	1.08(0.9,1.27)	1.08(0.92,1.27)	0%	1.00	0.90(0.69,1.16)	0.87(0.70,1.09)	0%	0.23	1.21(0.89,1.57)	1.07(0.76,1.50)	0%	—
Musculoskeletal AEs	0.76(0.35,1.21)	0.77(0.48,1.25)	63%	0.46	1.25(0.52,2.55)	1.07(0.72,1.58)	0%	0.73	0.72(0.48,1.08)	0.20(0.07,0.61)	—	—
Infection	0.84(0.63,1.08)	0.81(0.66,0.99)	20%	0.71	1.19(0.8,1.62)	1.30(0.98,1.73)	0%	0.76	0.75(0.49,1.17)	1.47(0.36,5.97)	66%	—
Skin AEs	1.53(0.77,2.44)	1.39(0.65,2.99)	0%	1.00	0.71(0.5,0.86)	0.84(0.54,1.30)	0%	1.00	2.14(1.24,3.25)	—	—	—
Peripheral edema	2.22(0.95,5.11)	1.05(0.74,1.48)	0%	0.37	2.00(0.59,4.9)	1.67(0.48,5.79)	52%	0.31	1.50(0.35,4.38)	3.73(0.77,18.14)	—	—

NM, network meta-analysis; PM, pairwise meta-analysis; I^2^, percentage of heterogeneity; PB, publication bias (*p* value of Beggs’ test); AEs, adverse effects; CNS, central nerve system.
